# Skeletal and cardiac muscle calcium transport regulation in health and disease

**DOI:** 10.1042/BSR20211997

**Published:** 2022-12-12

**Authors:** Mark A. Valentim, Aditya N. Brahmbhatt, A. Russell Tupling

**Affiliations:** Department of Kinesiology and Health Sciences, University of Waterloo, Waterloo, Ontario, N2L 3G1, Canada

**Keywords:** muscle, NCX, phospholamban, sarcolipin, sarcoplasmic reticulum, SERCA

## Abstract

In healthy muscle, the rapid release of calcium ions (Ca^2+^) with excitation–contraction (E-C) coupling, results in elevations in Ca^2+^ concentrations which can exceed 10-fold that of resting values. The sizable transient changes in Ca^2+^ concentrations are necessary for the activation of signaling pathways, which rely on Ca^2+^ as a second messenger, including those involved with force generation, fiber type distribution and hypertrophy. However, prolonged elevations in intracellular Ca^2+^ can result in the unwanted activation of Ca^2+^ signaling pathways that cause muscle damage, dysfunction, and disease. Muscle employs several calcium handling and calcium transport proteins that function to rapidly return Ca^2+^ concentrations back to resting levels following contraction. This review will detail our current understanding of calcium handling during the decay phase of intracellular calcium transients in healthy skeletal and cardiac muscle. We will also discuss how impairments in Ca^2+^ transport can occur and how mishandling of Ca^2+^ can lead to the pathogenesis and/or progression of skeletal muscle myopathies and cardiomyopathies.

## Introduction

Calcium (Ca^2+^) is a divalent cation which is indispensably involved with molecular signaling. Innately, Ca^2+^ has flexibility in its bonding angles and lengths, allowing for an array of potential ligation patterns [1]. The diversity in bond arrangements allows molecules to present binding sites with numerous variations. These slight differences among binding sites allow the kinetics of bond formation to vary among Ca^2+^-binding molecules [1,2]. Thus, the complexity of Ca^2+^ signaling becomes apparent as it can be regulated by the location and expression of the Ca^2+^-binding proteins and the kinetics of the Ca^2+^-binding site.

Within myofibers, cytosolic Ca^2+^ concentrations ([Ca^2+^]_cyt_) can fluctuate from resting concentrations of 100 nM to values above 1000 nM during tetanus [3]. When [Ca^2+^]_cyt_ is elevated above resting concentrations, Ca^2+^ interacts with two categories of Ca^2+^ binding molecules: buffers and sensors [4]. Ca^2+^ sensors elicit a downstream signal when binding occurs. The temporal range of Ca^2+^ signaling can be as brief as seconds but may also exist on the timescale of days [5]. Ca^2+^ signaling is effectively diminished when [Ca^2+^]_cyt_ reverts back to resting concentrations. One strategy for increasing the rate of [Ca^2+^]_cyt_ decay is through Ca^2+^ buffering. Buffers act to sequester Ca^2+^ without being directly incorporated into molecular signaling. The presence of Ca^2+^ buffers allows for the regulation of Ca^2+^ diffusion, which can indirectly affect molecular signaling pathways [6–8].

Although Ca^2+^ buffering does have a role in altering intracellular Ca^2+^ transients (ICT), the decay of [Ca^2+^]_cyt_ is mediated primarily through the movement of Ca^2+^ across phospholipid membranes [1]. Within striated muscle, the main strategy for lowering [Ca^2+^]_cyt_ is through Ca^2+^ sequestration in the sarcoplasmic reticulum (SR), a membrane bound organelle which surrounds the contractile myofilaments [3]. Ca^2+^ is transported against a concentration gradient into the SR in an ATP-dependent manner. This occurs through the sarco(endo)plasmic reticulum Ca^2+^-ATPase (SERCA), which, under ideal conditions, will transport two Ca^2+^ for every ATP hydrolyzed [9,10]. SERCA not only has a critical role in the regulation of [Ca^2+^]_cyt_ but also in the regulation of the energy expenditure of the cell [11]. Within cardiac tissue, a smaller, yet notable proportion of the cytosolic Ca^2+^ is also extruded across the plasma membrane to effectively reduce [Ca^2+^]_cyt_ during the cardiac cycle [12]. The role of Ca^2+^ transport is essential for the homeostatic function of cells, especially within excitable, contractile tissue. During dysregulation, inadequate control of [Ca^2+^]_cyt_ can result in the unwanted activation of proteolytic and apoptotic pathways, leading to muscle damage, dysfunction, and even disease. In this review, we discuss the role of Ca^2+^ transport in the maintenance of healthy muscle as well as the role it can have in the genesis and exacerbation of pathological states.

## Regulation of calcium transport in healthy muscle

As voluntary tissue, force generation by skeletal muscle follows the excitation of the sarcolemmal membrane by an associated motor neuron [13]. Neural signaling results in depolarization of the myofiber plasma membrane [13], which is detected by the voltage sensitive dihydropyridine receptors (DHPR) located within the transverse tubule (T-tubule) membrane [14]. In skeletal muscle, the DHPR is physically linked with the ryanodine receptor (RyR) and when changes in voltage are detected, the DHPR acts to increase the open probability of the RyR leading to increased Ca^2+^ release [15]. In cardiac muscle, SR Ca^2+^ release is slightly different such that when Ca^2+^ enters the cytosol via the DHPR channels, it binds to and opens RyR channels through a mechanism referred to as Ca^2+^-induced Ca^2+^ release (CICR) [12].

The RyR is a Ca^2+^ channel embedded in the terminal cisternae of the SR that functions as the major Ca^2+^ release channel inside muscle cells and, when activated, increases the [Ca^2+^]_cyt_ [15]. Within healthy muscle tissue, the increase in [Ca^2+^]_cyt_ results in the immediate activation of SERCA, which acts to pump Ca^2+^ back into the SR. However, with the continuance of high frequency neural signaling the rate of Ca^2+^ outflow is greater than the ability for SERCA to re-sequester Ca^2+^. Consequently, [Ca^2+^]_cyt_ rises within the myofiber and binds to troponin C resulting in the movement of tropomyosin and thus uncovering the myosin binding site on the thin filaments [16,17]. With the myosin binding site exposed, crossbridge formation between the thick and thin filaments results in the generation of force [18,19]. Muscle relaxation will not occur until the termination of high frequency neural signaling and inactivation of RYR. Upon the cessation of Ca^2+^ release, the decay phase of [Ca^2+^]_cyt_ begins.

In a single-twitch stimulus, a rapid rise in [Ca^2+^]_cyt_ to a peak is quickly followed by a decay, all within milliseconds [20–22]. The decay phase of [Ca^2+^]_cyt_ has a negative exponential relationship in which the initial rate of decay is rapid but as [Ca^2+^]_cyt_ approaches pre-stimulation resting concentrations the rate of calcium uptake is attenuated. Within skeletal muscle tissue, the characteristics of a Ca^2+^ transient varies across different types of muscle fibers. Baylor and Hollingworth (2003) compared the ICTs of slow fibers from soleus tissue and fast fibers from EDL muscle [23]. They found identical times to peak [Ca^2+^]_cyt_ during Ca^2+^ release in the slow and fast fibre types; however, the peak [Ca^2+^]_cyt_ amplitude was two times greater and the ICT half duration was ∼1.6 times shorter in fast EDL fibers compared with the slow soleus fibers [23]. The rate of Ca^2+^ sequestration in the final half of the ICT decay phase was also three times greater in fast fibers compared with slow fibers [23]. The fiber type differences in the rate of ICT decay are believed to contribute to different force summation responses at submaximal stimulation frequencies. With evoked contractions at 67 Hz, the ICT amplitudes and force grew only slightly with continued stimulation in fast fibers whereas ICT amplitude and force increased with each subsequent stimulation in slow fibers [23].

**Figure 1:**
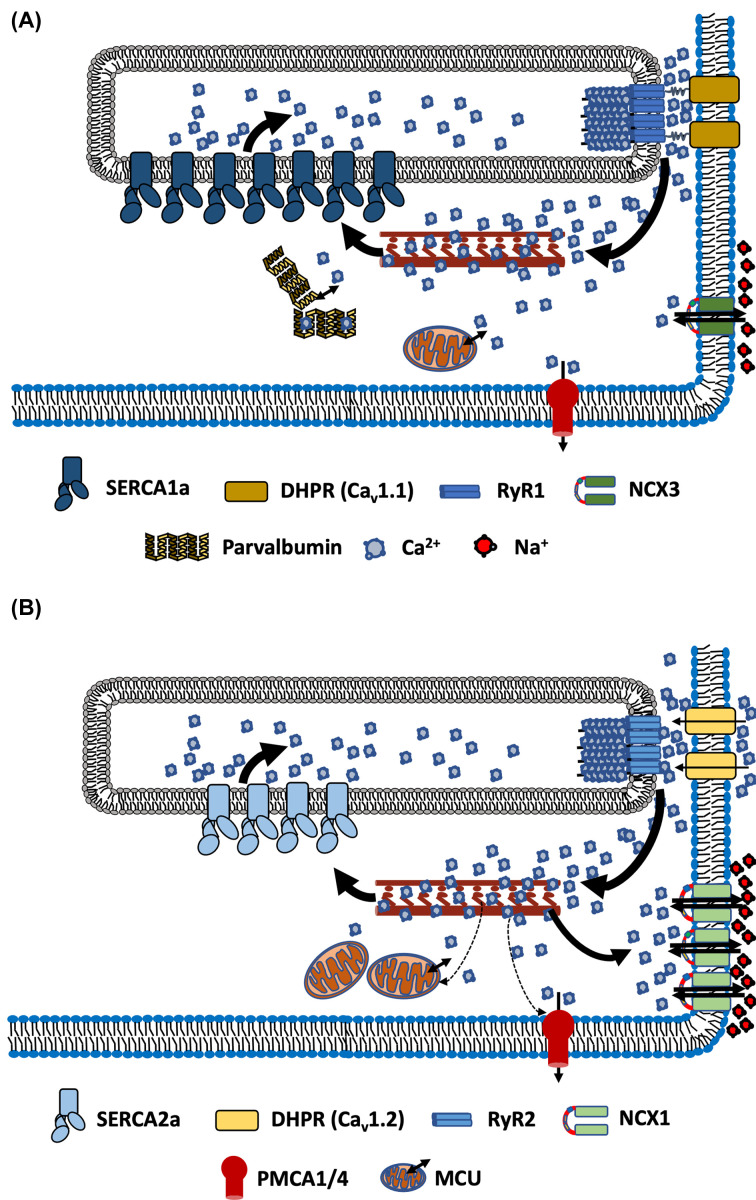
Movement of Ca ^2+^ in skeletal myofibers and cardiomyocytes during E-C coupling Movement of Ca^2+^ in skeletal myofibers (**A**) and cardiomyocytes (**B**) during E-C coupling. The relative contribution of Ca^2+^ transport proteins to Ca^2+^ ion removal during the decay phase of Ca^2+^ transients is indicated by different arrows with thicker solid arrows indicating a major contribution, thinner dashed arrows indicating a minor contribution, and no contribution of expressed proteins where arrows are not shown. Refer to the text for further details.

The myocardium of the heart contracts in response to neural excitation to pump blood out of the heart and relaxes upon the cessation of neural stimulation to allow the heart to refill with blood. Cardiomyocytes within the myocardium express similar but different isoforms of Ca^2+^ handling proteins than found in skeletal muscle as discussed below; however, their regulation and contribution to excitation–contraction (E-C) coupling vary slightly (see Figure 1). Like skeletal muscle, SERCA is the dominant Ca^2+^ transport protein in cardiomyocytes that contributes to the decay of Ca^2+^ transients during E-C coupling [12,24]. Unlike skeletal muscle, the NCX and the slow Ca^2+^ sequestering systems, which include mitochondrial Ca^2+^ uniport (MCU) and sarcolemmal Ca^2+^ ATPase (PMCA), also contribute to Ca^2+^ decay in cardiac muscle [12,24]. During cardiomyocyte relaxation in rat ventricle, SERCA accounts for as much as 92% of ICT decay, while that of NCX, and the slow system contribute 7% and 1%, respectively [25]. In contrast, within rabbit ventricle myocytes incubated at 25°C, the contribution from SERCA, NCX, and the slow systems has been reported as 70%, 27%, and 3%, respectively [26]; however, under stimulated conditions (in the presence of isoproterenol), the contribution by SERCA has been found to be increased to 83% in rabbits [27].

### Sarco(endo)plasmic reticulum Ca^2+^-ATPase

SERCA is a 110 kDa P-type ATPase, which is embedded in the membrane of the longitudinal SR. Structurally, SERCA is composed of three cytoplasmic domains: phosphorylation (P-domain), actuator (A-domain) and nucleotide-binding (N-domain); and 10 transmembrane helices (M1–M10) [28]. During the catalytic cycle, SERCA undergoes drastic conformational changes with the binding of cations and ATP by alternating between an E1 and E2 state [29]. Previous genomic and proteomic analyses have shown that there are multiple SERCA isoforms derived from three distinct genes: ATP2A1, ATP2A2, and ATP2A3. From these genes, 13 different mRNA splice variants and 10 protein isoforms have been identified [30–34]. SERCA isoforms are between 75 and 84% homologous [35]. The SERCA isoform expression changes with development and aging and varies between tissues. Within fetal and neonatal rat skeletal muscle, SERCA1b and SERCA2a are expressed in fast fibers while SERCA2a is expressed in slow fibers [35]. In rat heart, SERCA2a and SERCA3 are initially expressed during early developmental stages but as development continues only SERCA2a mRNA remains expressed [35]. In adult rats, SERCA1a becomes most commonly found within fast twitch muscle fibers, and SERCA2a is found most commonly in slow twitch fibers and myocardial tissue [35]. Smooth muscle expresses both SERCA2a and SERCA2b [35,36]. Nonmuscle tissue is known to express SERCA2b and SERCA3a–c [35].

Despite considerable homology among SERCA isoforms, there are drastic differences in the lusitropic measures between these tissue types. Differences do exist in the kinetics of SERCA isoforms [37]; however, the large contrast in relaxation appears to be more so associated with differences in the quantity of SERCA molecules expressed, with fast twitch fibers expressing ∼5-fold greater levels of SERCA compared with slow twitch fibers [11,38]. Another factor affecting ICT decay differences among fiber types is the expression of the SR luminal protein calsequestrin, which is greater in type II muscle fibers [38]. Greater calsequestrin expression results in more Ca^2+^ buffering capacity within the SR resulting in less back-inhibition while SERCA pumps Ca^2+^ [38]. Thus, with greater Ca^2+^ buffering within the SR lumen, SERCA can maintain a higher rate of Ca^2+^ uptake [38].

The major isoform of SERCA that is expressed in cardiac tissue is SERCA2a with greater expression in the atria compared with ventricles [39], which is associated with faster contractile kinetics of atria compared with ventricles [40]. The ventricles of the heart are also thicker and produce stronger contractions than atria [39] and considering the importance of SR Ca^2+^ load to contractile force, SERCA2a expression and function is vital in these chambers. Studies in transgenic animal models provide good evidence for this. In animal models with reduced SERCA2a activity, the contractility of the left ventricle (LV) is significantly impaired compared with the wild-type controls [41]. Conversely, in models with increased SERCA2a activity, contractility of the LV is significantly enhanced [42].

### Phospholamban

**Figure 2:**
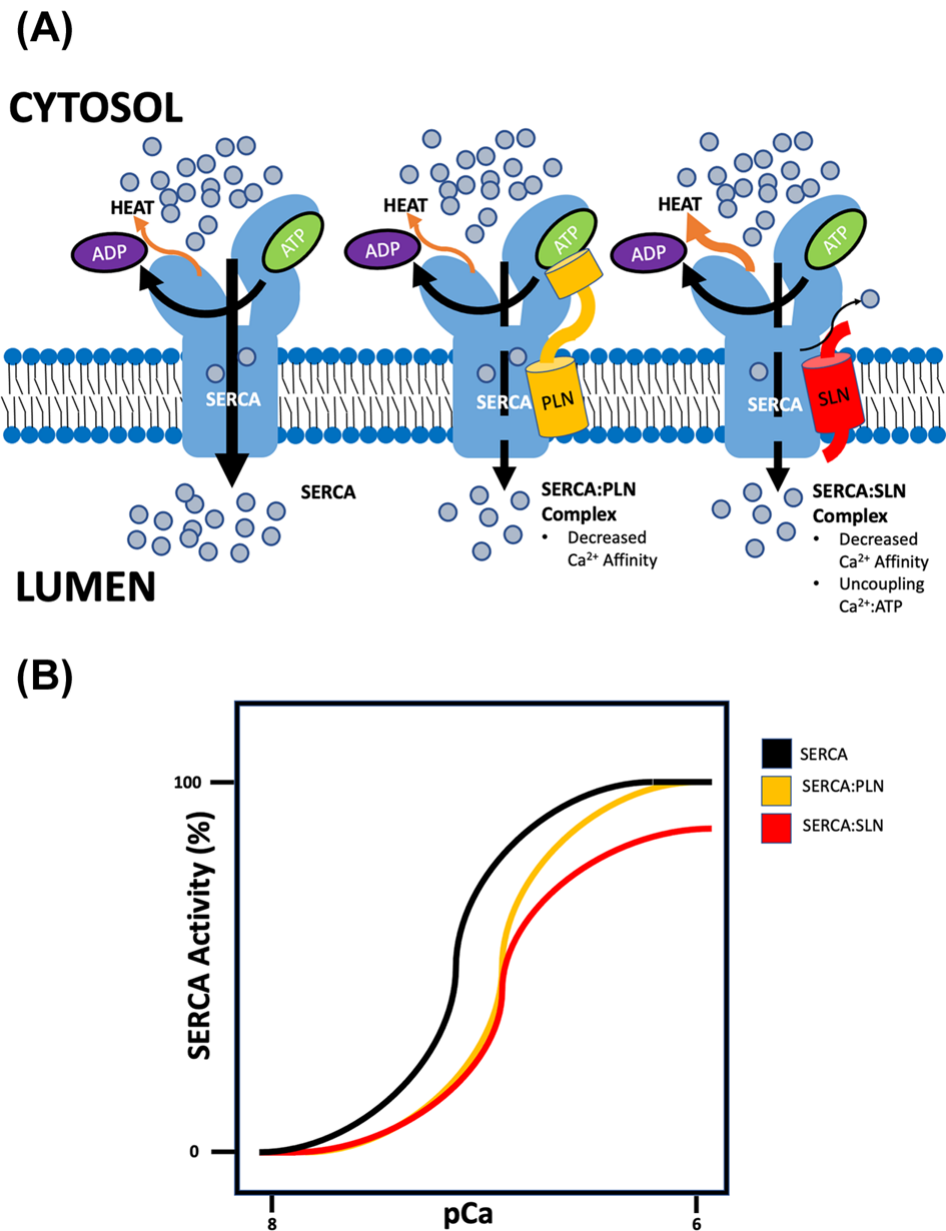
Regulation of SERCA by PLN and SLN (**A**) A cartoon model showing a SERCA molecule with no protein inhibitors bound (left), PLN bound (middle), and SLN bound (right) illustrates the physical and functional interaction between SERCA and its endogenous protein regulators. (**B**) A graphical summary shows the independent effects of PLN and SLN on SERCA activity. Refer to the text for further details.

Several proteins have been identified which act to regulate SERCA function either positively or negatively [43–45]. Among these SERCA regulators, phospholamban (PLN) is one of the most studied due to its vital role in cardiac function and disease [46–48]. PLN is expressed in cardiac tissue and in type I fibers of skeletal muscle [49,50], where it predominantly associates with SERCA2a [44]. However, it should be noted that work from our group has identified that in human muscles PLN can be found in type II fibres as well [44]. PLN is a 52-amino acid protein located in the SR membrane where it can interact with SERCA (Figure 2) [51,52]. PLN is composed of a small luminal domain, a transmembranous domain and a cytosolic domain [46,53,54]. The transmembranous domain consists of a single helix, which can directly bind to the Ca^2+^-binding sites formed by the M2, M4, M6 and M9 helices of SERCA [53,55]. In binding to SERCA, PLN elicits an inhibitory effect on the Ca^2+^ pump by reducing the apparent affinity of Ca^2+^ binding [56,57]; however, at maximal [Ca^2+^]_cyt_, PLN dissociates from SERCA and does not affect *V*_max_ [58–60], but the mechanism behind this remains to be elucidated.

The inhibitory action of PLN on SERCA can be disrupted via phosphorylation of two sites within the cytosolic domain of PLN. These phosphorylation sites are targeted by two different kinases: Ser16 by cAMP dependent kinase A, and Thr17 by Ca^2+^ calmodulin dependent kinase II (CAMKII) [46,61]. Upon phosphorylation of either of these sites, PLN will dissociate from SERCA while remaining in the SR membrane either in its monomeric or homopentameric form [61]. PLN phosphorylation acts as a mechanism for the cell to quickly alter the rate of Ca^2+^ sequestration. During times where [Ca^2+^]_cyt_ is elevated, CAMKII will detect these elevations and respond by phosphorylating PLN, thus relieving its inhibitory effect on SERCA and allowing for faster calcium clearance [62]. PLN inhibition is also alleviated by β-adrenergic stimulation through increasing the kinase activity of PKA [46,62–64]. This is especially important in cardiac tissue when the work demand increases such as during exercise. Exercise elicits an increased oxygen demand of the recruited skeletal muscles. In order to ensure adequate oxygen delivery to meet the demands of the recruited muscles, both heart rate and cardiac contractility must increase to elevate cardiac output. With increases in the rate of cardiac cycling, the rate of Ca^2+^ removal from the cytosol must also increase to ensure adequate relaxation of the myocardium during diastole [27]. In return, this would contribute to increased SR Ca^2+^ load and thus greater Ca^2+^ release and systolic force in subsequent beats [46,63]. The ability to modulate the inhibitory effect of PLN on SERCA is an important mechanism to quickly alter the rate of calcium sequestration, which may be necessary depending on the situational needs of the contractile tissue.

### Sarcolipin

Another SERCA regulatory protein, which has garnered a lot of recent interest is sarcolipin (SLN). SLN is also a transmembrane protein found with the SR membrane (Figure 2). Structurally, it contains an 8 amino acid cytoplasmic domain, a 19 amino acid transmembrane alpha helix, and a 4 amino acid luminal tail [57,65–67]. SLN shares considerable homology with PLN within the transmembrane helix and thus associates with SERCA in a similar manner [46,68]. In humans, SLN is most abundantly expressed in fast-twitch type IIA muscle fibers where it most commonly associates with SERCA1a, although a small percentage of slow-twitch type I fibers also co-express SLN and SERCA1a [44]. Seemingly contradictory, muscles with the highest reported SLN expression in mice are the slower oxidative muscles (i.e soleus, red gastrocnemius, and diaphragm) [49,69,70]. This is likely explained by the fact that these slow oxidative mouse muscles contain a fiber type distribution in which 40–50% of fibers are type IIA [71]. Thus, like in human skeletal muscle, it appears SLN may be expressed within mouse type IIA fibers where it can regulate SERCA1a; however, single fiber data from mice are required to confirm this. SLN is also highly expressed in atrial cardiomyocytes where it regulates SERCA2a function, at least in mice [69,72].

Like PLN, SLN is an inhibitory regulator of SERCA, reducing the apparent affinity of cytosolic Ca^2+^ with SERCA. Unlike PLN, SLN binding can reduce the maximal catalytic activity of SERCA [44,57]. SLN has also been identified for its thermogenic properties [73]. SLN remains bound to SERCA during its catalytic cycle and interacts with one of the SERCA-Ca^2+^ binding sites [74]. Consequently, SLN but not PLN can reduce the efficiency of Ca^2+^ transport [74–76]. When this uncoupling of Ca^2+^ pumping occurs, more ATP must be consumed to pump the same quantity of Ca^2+^. Research has shown that this innate uncoupling mechanism is involved in the maintenance of core body temperature during cold exposure and energy balance during caloric surplus [73,75,77,78]. However, it should be noted that thermogenesis is also regulated through Ca^2+^ cycling mechanisms in resting mammalian muscles that don’t express SLN [79]. Additionally, the uncoupling mechanism elicited by SLN may also influence Ca^2+^ signaling as previous work from our laboratory has shown that SLN expression can affect calcineurin activity in models of muscle overload, disease and disuse [71,80–82]. Further evidence for the role of SLN in Ca^2+^ signaling has been shown by Maurya and colleagues (2017), who found that changes in SLN expression can affect the activity of the transcriptional coactivator, peroxisome proliferator-activated receptor-gamma coactivator-1alpha (PGC1α), CAMKII, and mitochondrial biogenesis pathways [83].

### Other emerging SERCA regulators

Although PLN and SLN are the two most researched SERCA regulatory proteins, recent bioinformatics studies have identified other proteins with a similar helical pattern, which could potentially also regulate SERCA. Of the proteins identified, dwarf opening reading frame (DWORF) and myoregulin (MLN) appear to have gained the most interest [84,85]. Unlike negative regulators such as PLN and SLN, DWORF is believed to be a positive regulator of SERCA. By binding to SERCA, DWORF prevents the binding of inhibitory SERCA regulators such as PLN and SLN [45]. In doing this, DWORF maintains the sensitivity of SERCA at low [ Ca^2+^]_cyt_. MLN, on the other hand, is believed to be another inhibitory regulator of SERCA [43]. Interestingly, MLN also appears to have multiple sites, which could be phosphorylated like that of PLN [43]. More studies are needed to fully understand the function of these proteins *in vivo*.

### Redox regulation of SERCA

Redox signaling plays an important role in the regulation of several of the major physiological systems of muscle including the SR Ca^2+^ regulatory system [86]. SERCAs are redox-sensitive proteins that may be activated by low levels of reactive oxygen (ROS) and nitrogen (RNS) species [87–89]. Physiologically, activation of SERCA2b in vascular smooth muscle occurs through redox signaling where nitric oxide and superoxide anion, through the formation of peroxynitrite, activate SERCA2b by reversible S-glutathiolation on Cys674 resulting in arterial relaxation [87]. The same molecular mechanism is also involved in the activation of SERCA2a in cardiac myocytes by the nitric oxide derivative nitroxyl, which may require an interaction with oxidized PLN [89]. Our work showing that glutathione depletion and cellular oxidation increased SERCA2a content, and maximal Ca^2+^-ATPase activity in rat diaphragm [88] supports the view that SERCA pump activity in skeletal muscle is also regulated through redox signaling.

### Na^+^/Ca^2+^ exchanger

As mentioned, the NCX plays an important role in myocardial Ca^2+^ transport. Lying on the sarcolemma of cardiomyocytes, NCX is responsible for controlling the exchange of 1 Ca^2+^ ion for every 3 Na^+^ ions across the membrane [12,90–92]. The direction of this exchange can either be forward (Ca^2+^ out and Na^+^ in) or reverse (Ca^2+^ in and Na^+^ out) depending on the electrochemical gradient across the membrane [12,90–92]. When the membrane is depolarized, initially the reverse mode is favored because the membrane potential is greater than the combined (Na^+^ and Ca^2+^ ions) equilibrium potential of NCX, but the amount of Ca^2+^ entry is <1 μM [12,92]. As the [Ca^2+^]_cyt_ rises due to SR Ca^2+^ release, the membrane potential effect becomes negligible, and the direction of the exchanger now changes to the forward mode with Ca^2+^ being extruded from the cytosol [12,92]. Although small changes in the subsarcolemmal [Na^+^] and [Ca^2+^] can impact the direction of the exchanger, under physiological conditions, the direction is largely driven by the [Ca^2+^]_cyt_ [92]. After the release of Ca^2+^ into the cytosol during a contraction, the flow of ions observed in the NCX will be inward, which will result in the entry of Na^+^ and the extrusion of Ca^2+^ from the cell [12,91]. The opposite will occur with a positive membrane potential or increased cytosolic Na^+^ levels [12,92]. Under physiological conditions, NCX will be largely in its inward state [12]. Ion transport by NCX works in a similar stepwise fashion to SERCA, with ion binding sites facing the cytoplasmic side in the E1 state and facing the extracellular side in the E2 state [93–96].

NCX is regulated by Na^+^, Ca^2+^, and H^+^. Regulation by Na^+^ is referred to as Na^+^- dependent inactivation and this occurs in the presence of elevated intracellular Na^+^ levels [91,93,97]. High cytoplasmic Na^+^, such as during depolarization of the cell, leads to Na^+^ ions binding to their site on NCX in the E1 state, which then switches to an inactivated E1 state instead of cycling to an E2 state [93,95] This method of regulation is modulated by a part of the exchanger within the large cytoplasmic loop domain known as the XIP region as certain mutations within this region have been shown to abolish Na^+^-dependent regulation [97–99]. Ca^2+^ ions, on the other hand, up-regulate NCX activity by interacting with two other sites on the large cytoplasmic loop domain referred to as the Ca^2+^ binding domains 1 and 2 (CBD1 and CBD2) [91,100,101]. Although both CBD1 and CBD2 are involved in Ca^2+^-dependent activation, CBD1 is the primary Ca^2+^ sensor due to its 7-fold higher affinity for Ca^2+^ compared with CBD2, binding up to 4 Ca^2+^ ions at a range of 200 nM to 1 μM [101]. Upon a rise in cytosolic Ca^2+^ such as during E-C coupling, CBD1 and CBD2 change conformation and undergo an electrostatic shift bringing them closer together [101]. This conformational change allows the signal to be relayed via a small domain, called the α-catenin-like domain (CLD), to the transmembrane domain to increase NCX activity [100,101]. CBD2 can bind up to two Ca^2+^ ions during times of very high [Ca^2+^] to potentially help to overcome Na^+^-dependent inactivation by increasing the electrostatic potential [101]. Finally, intracellular pH modulates activity of NCX by increasing or decreasing its activity if the pH is increased or decreased, respectively [91,102–105]. When intracellular pH decreases, NCX inhibition occurs in two steps: (1) primary or fast blockade that occurs with rising H^+^ levels and works independent of the [Na^+^]_cyt_ levels and (2) secondary or slow blockade that is dependent on [Na^+^]_cyt_ levels, which enhance the affinity of NCX for H^+^ [102,103,105]. The mechanism for pH modulation of NCX was previously thought as H^+^ competing with Ca^2+^ for sites at CBD1 [91]. However, mutagenesis studies have identified histidine residues 124 and 165 as two important players which modulate NCX function allosterically through H^+^ binding, which is distinct from regulation by Na^+^ or Ca^2+^ [104].

Within skeletal muscle, two isoforms of NCX are expressed, NCX1 and NCX3 [106], with the latter being the predominant isoform [107]. Although NCX is expressed in skeletal muscle, it doesn’t appear to be directly involved in E-C coupling [108]. The rate of Ca^2+^ transport by the skeletal muscle NCX has been reported to be ∼30 times slower compared with cardiac muscle [109]. It has been postulated this transporter is more involved in regulation of [Ca^2+^]_cyt_ during repeated muscle contractions when [Ca^2+^]_cyt_ is very high [109,110]. Furthermore, NCX3 knockout mice display low levels of necrosis within the muscle fibers, suggesting that the NCX is important for maintenance of Ca^2+^ homeostasis within skeletal myofibers [107].

### Calcium buffering proteins

The presence of cytosolic Ca^2+^ buffering proteins can also influence the decay phase of Ca^2+^ transients in muscle. Within the cytosol of myofibers, the most well documented Ca^2+^ buffering protein is parvalbumin (PV), which has a role in lowering [Ca^2+^]_cyt_ [23,111,112]. Within small mammalian animal models such as mice and rats, PV has been found in neural tissue and type II muscle fibers [113]. In contrast, little or no PV is expressed in cardiac and type I muscle fibers [111–113]. Furthermore, PV has not been detected in human skeletal muscle [114]. PV is a 12 kDa protein that binds Ca^2+^ at an optimal ratio of 2 mol Ca^2+^:1 mol PV [115,116]. The two high affinity binding sites are occupied by Mg^2+^ at resting [Ca^2+^]_cyt_ (<100 nM) [117]. The rate at which PV can act to sequester Ca^2+^ is dependent on the rate of dissociation of Mg^2+^ from the binding sites [112,118]. Consequently, PV buffering does not occur immediately following rises in [Ca^2+^]_cyt_, which is why it is often considered a slow Ca^2+^ buffer [117]. The binding kinetics of PV explain why its increased expression has been shown to reduce 1⁄2 relaxation time and increase the rate of relaxation (−d*f*/d*t*) without altering the single twitch contractile properties of skeletal muscle [6,7,112,119,120]. However, during low frequency stimulation (30 and 50 Hz) of type I fibres, PV overexpression attenuates force production [7]. Taken together, this research provides evidence that PV is a Ca^2+^ buffering protein, which can increase rates of Ca^2+^ sequestration during E-C coupling.

Although the mitochondria are not traditionally known for Ca^2+^ buffering, there is evidence showing that Ca^2+^ uptake through the mitochondrial Ca^2+^ uniporter is important for the maintenance of myocyte homeostasis [121]. Although it’s fairly well established that mitochondrial Ca^2+^ uptake in cardiomyocytes does not alter [Ca^2+^]_cyt_ transients or cardiac contractility, there is evidence that mitochondria can modulate [Ca^2+^]_cyt_ in fast-twitch skeletal muscle fibers under certain conditions [121], a view that is supported by recent evidence indicating that mitochondria in fast-twitch mouse fibers have a high Ca^2+^-buffering capacity [122]. A recent paper by Butera and colleagues (2021) highlighted the dynamic relationship between the mitochondria and PV regarding Ca^2+^ buffering [123]. Butera and colleagues reported that when PV was overexpressed, mitochondrial volume and number decrease but when PV was down-regulated, the mitochondria increased in size and quantity [123]. This alone may not indicate that mitochondria are being up-regulated to buffer Ca^2+^ but rather mitochondrial biogenesis results from the activation of Ca^2+^-dependent signaling pathways in the absence of PV. However, an interesting observation made by Butera and collogues was that when PV was down-regulated there was a significantly greater proportion of mitochondria located near Ca^2+^ release units suggesting mitochondria may have a larger role in Ca^2+^ buffering than originally thought. Another interesting consideration is that there is a grouping of mitochondria localized around the longitudinal SR, where SERCA is located [124]. If mitochondria do have a role in buffering Ca^2+^, then the location of the mitochondria may have a role in altering Ca^2+^ concentrations in areas around SERCA [123,125].

## Calcium transport in diseased muscle

### Calcium related myopathies without muscle wasting

Among skeletal muscle myopathies, there is a grouping that involves prolonged contractions due to a reduced ability to lower ICTs during the relaxation phase of E-C coupling. Most notably among this grouping of myopathies is Brody’s disease and Brody’s syndrome, both of which involve reductions in SERCA1a activity [126]. Although similar in name and presentation, Brody’s disease but not Brody’s syndrome involves a mutation in the ATP2A1 gene encoding SERCA1a [127]. Even within the myopathies classified as Brody’s disease, there is a degree of heterogeneity in the ATP2A1 mutations as they have been documented in the A-, P-, and N-domains as well as several others at various points along the transmembrane helices [126]. Among the genetically unresolved cases of Brody’s syndrome, one possibility is that mutations of a SERCA inhibitor (e.g., SLN, PLN, or MLN) could be causing reductions in SERCA function [128], but none have been documented to date.

Although there are differences in the mutations leading to myopathy pathogenesis of Brody’s disease and Brody’s syndrome, the symptoms of these diseases are similar in presentation. Research has shown that reductions in SERCA activity appear to be specific to the SERCA1a isoform in type II muscle fibers [129]. As a result, prolonged contractions with delays in relaxation become apparent during faster contractions, which require the activation of type II fibres. Sustained contractions in this disease have been associated with increased plasma creatine kinase, cases of rhabdomyolysis and malignant hyperthermia-like episodes [126,130,131]. Despite these acute harmful effects brought on by the reduced rate of skeletal muscle relaxation, there does not appear to be a muscle wasting aspect to these group of diseases [132]. Although some reports have suggested atrophy, which is likely a result of the underutilization of the type II fibres [126], there does not appear to be progressive wasting brought on by this disease.

Immunohistochemical analysis of muscle samples from Brody’s disease patients revealed a severe reduction in the SERCA content of type II fibres while the SERCA content in type I fibres was normal [126]. Furthermore, immunoblot quantification from whole muscle homogenates revealed an absolute reduction in SERCA1a content [126]. Within human tissue it has been shown that type II fibres do express small amounts of the SERCA2a isoform [44,133]. This may explain why Ca^2+^ concentrations are able to return to resting levels in type II fibres even when the SERCA1a protein is either partially functioning or completely nonfunctional. However, since the endogenous expression of SERCA2a in type II fibres is relatively low, in Brody’s disease a substantially smaller quantity of SERCA pumps contributes to Ca^2+^ sequestration, thus resulting in a slower decay of the ICT.

Pan and colleagues attempted to create a murine model of Brody’s disease by knocking out the gene encoding SERCA1 [134]. In creating this model, it was found that neonatal mice were born healthy but became cyanotic and died shortly after birth [134]. It was hypothesized that the SERCA1 knockout mice pups were unable to properly ventilate due to the relatively high proportion of type II fibers in mouse diaphragm resulting in a greater reliance on SERCA1a for diaphragm relaxation compared with humans.

### Myopathies involving elevations in resting calcium concentrations

Another grouping of muscle diseases is characterized by muscle wasting. These diseases share a commonality in that elevations in resting Ca^2+^ concentrations appear to contribute to the pathogenesis, and in some cases, progression of the disease phenotype. These diseases include Duchenne’s muscular dystrophy (DMD) and centronuclear myopathy (CNM).

Muscular dystrophies are a heterogenous group of inherited disorders, which vary genetically and in clinical presentation [135–137]. These disorders involve increased muscle turnover resulting in progressive atrophy of the skeletal muscles [137–139]. Among these diseases, DMD is considered the most common and the most severe [139]. DMD is an X-linked recessive disease which results in atrophy of the limb, axial, and facial muscles. The life expectancy of individuals with this disease is reduced by about 75% at which point death is usually caused by respiratory or cardiac complications [139].

From a molecular standpoint, DMD arises from mutations in the *DMD* gene resulting in a deficiency in the dystrophin protein. Dystrophin is a rod-shaped cytoskeletal protein which forms a critical link between the submembrane cytoskeleton and proteins of the extracellular matrix. Importantly, dystrophin associates with proteins within the sarcolemma, which are integrated in pathways associated with Ca^2+^ influx and ROS signaling. Muscular dystrophy affects fibres specialized for faster contractions, as the instability from the absence of dystrophin results in greater myofiber damage [140]. The myofiber damage results in a continuous cycle of muscle turnover through the up-regulation of degeneration and regeneration pathways [141,142]. The increased muscle turnover subsequently results in inflammation and the infiltration of collogen into the muscle making it more fibrotic [141,143,144].

There is still some ambiguity in how the deficiency of dystrophin results in the progression of DMD. Currently, there are two leading theories as to how DMD progresses, both of which involve elevations in [Ca^2+^]_cyt_. The first theory posits the lack of dystrophin results in transient tearing of the sarcolemma membrane during contractions. The membrane damage could allow for an influx of extracellular Ca^2+^ into the myofiber [145–147]. A second theory lies in dystrophin’s role as a scaffolding protein and its role in positioning membrane ion channels. In the absence of dystrophin, these ion channels may function improperly and increase Ca^2+^ influx into the myofiber [145–147]. Prolonged elevations in [Ca^2+^]_cyt_ can increase proteolytic activity and the production of ROS [82,148–150]. Furthermore, increased ROS can inactivate SERCA function which can further augment Ca^2+^ dysregulation [88,151–155]. Previous work by Goonasekera and colleagues (2011) has shown that elevations in [Ca^2+^]_cyt_ alone are enough to promote a shift toward a DMD like phenotype [156]. This was shown through preventing increases in [Ca^2+^]_cyt_ by overexpressing SERCA in dystrophic tissue, which ameliorated the pathology [156]. This work has provided strong evidence for the role of Ca^2+^ in promoting the progression of DMD. Recently, the use of CDN1163, a pharmaceutical SERCA activator, has been used as a therapeutic intervention for DMD through its ability to promote Ca^2+^ sequestration in the SR. Specifically, Nogami et al. (2021) administered CDN1163 to *mdx* mice, which reduced [Ca^2+^]_cyt_ and decreased muscular degeneration and fibrosis [157]. Using a different approach to restore SERCA function in DMD models, Gehrig and colleagues (2012) demonstrated that both transgenic and pharmacological overexpression of the chaperone protein, heat shock protein 72 (HSP72), could attenuate the progression of DMD [158]. HSP72 associates with SERCA and acts to protect it against oxidative damage and inactivation [152,158,159].

CNM is a heterogeneous group of inherited neuromuscular diseases characterized by increased localization of centralized nuclei. Although variance exists among disease phenotypes, other common histological indicators include an increased proportion of type I fibres, centralized aggregations of oxidative activity, muscle atrophy and weakness [160]. The most severe cases of CNM arise in a X-linked inheritance pattern from a mutation in the *MTM1* gene. *MTM1* encodes for myotubularin, which functions to regulate PI(3)P, endocytosis and endolysosomal function [161–163]. Milder cases of CNM have been reported in individuals with mutations in *DNM2*, which encodes dynamin 2, and *BIN1*, which encodes amphiphysin-2 [163]. MTM1, DNM2, and BIN1 are all involved with membrane trafficking suggesting they all work through the same pathogenic pathway [161–166]. Interestingly, Ca^2+^ dysregulation is also a common feature within each of these genetic variations of CNM [163,167,168]. Recently, mutations in *RYR1* and *TTN*, the genes encoding skeletal muscle RyR and the cytoskeletal protein titin, respectively, have also been implicated in the development of a CNM phenotype providing greater evidence for the involvement of Ca^2+^ in CNM pathogenesis [169,170]. RyR1-related CNM differs from other forms of CNM as there appears to be no direct links to defective membrane trafficking [163]. Mutations to *TTN* which cause CNM are relatively heterogenous but most appear to involve C-terminus truncations that are associated with a reduction in calpain-3 and nebulin-2, two proteins which interact with the C-terminal region, which may cause irregularities in Ca^2+^ release [163,169,171].

While trying to characterize the role of PLN in SERCA regulation in skeletal muscle, Song and colleagues (2004) discovered that PLN overexpression specifically in type I muscle fibres results in muscle disease [172], which was later recognized as a CNM-like phenotype [173]. Fajardo et al. (2015) determined that mice with targeted overexpression of PLN in type I fibres displayed an increased centralization of nuclei and oxidative activity as well as type I fibre hypotrophy at 1 month of age [173]. Furthermore, a fibre type shift toward type I fibres was evident by 4–6 months [173]. Interestingly, because the *Pln* transgene is attached to the β-MHC promoter in this model, the shift to type I fibres leads to greater expression of PLN causing greater disease severity. In this model, there is also atrophy of type I fibres while there appears to be a compensatory hypertrophy of type II fibres [173]. Through histological analysis, it was suggested by Fajardo and colleagues that similarities in oxidative staining and fibrosis make PLN-related CNM resemble a phenotype which appears more closely related to TTN- and RyR-CNM [173]. Furthermore, Fajardo and colleagues reported a significant 53% reduction in SERCA activity and increases in both total and monomeric PLN content in muscle samples from three human CNM patients compared with five healthy subjects. Overall, this work has shown that overexpressing a SERCA inhibitor and severely impairing SR Ca^2+^ transport can cause CNM. Therefore, future studies should assess whether targeting SERCA function is a viable therapeutic strategy for this disease.

## Dysregulation of Ca^2+^ transport in heart disease

### Abnormal Ca^2+^ loading in heart failure

Heart failure (HF) can present with either a reduced (HFrEF) or preserved (HFpEF) ejection fraction [174,175]. HFrEF involves the inability of the LV to generate sufficient force to pump out enough blood due to cardiomyocyte loss and dysfunction (e.g., following a myocardial infarction) [174,176]. This leads to eccentric remodeling whereby the existing myocytes stretch and become thinner, alongside an increase in fibrosis in the extracellular space [174]. HFpEF involves the inability of the LV to sufficiently relax and allow for filling due to the enlargement of cardiomyocytes that is caused by an increase in afterload (e.g., hypertension) [174,176]. This leads to concentric remodeling where the walls of the LV thicken and elevate filling pressures [174]. Overall, in both cases, the LV walls enlarge causing dysfunction in the chamber [174–176].

It is widely observed from electrophysiological studies that myocytes from failing hearts have abnormal Ca^2+^ transients that are smaller in amplitude and longer in duration which result in abnormal force production [177–181]. It is well established that cardiomyocytes from nonfailing hearts display a positive force–frequency relationship with increasing frequencies of stimulation causing increases in systolic force [182–184]. However, in myocytes from failing hearts, this is only true at lower frequencies as force decreases at higher frequencies of stimulation (i.e., negative force–frequency relationship) and accordingly, the peak [Ca^2+^]_cyt_ is decreased in these cells [181,183–185]. In contrast, diastolic force increases with increasing frequency at all stimulation frequencies in the cells from failing hearts and this is associated with an increase in diastolic [Ca^2+^]_cyt_ [181,186].

While decreases in Ca^2+^ release with HF could be due to defective coupling between the RyR and DHPR, reduced SR Ca^2+^ content could also contribute. This was assessed in one study using a canine model of HF by assessing the CICR gain, which is the amount of Ca^2+^ released from the SR for a given level of current density from the DHPR channel and ε, which is the effectiveness of coupling between DHPR activation and SR Ca^2+^ release [178]. The CICR gain was lower in the failing cardiomyocytes along with lower SR Ca^2+^ content compared with the healthy cardiomyocytes, but no difference was observed for ε between the two groups. This indicates that in this model of HF it was the reduced SR Ca^2+^ content that led to reduced Ca^2+^ release in the failing cardiomyocytes and not reduced ε. Furthermore, after bathing the failing cardiomyocytes in a high Ca^2+^ solution (5 mmol/L) to bring the SR Ca^2+^ content back to levels observed in their healthy counterparts, CICR gain was recovered to levels seen in the healthy cells. This is further supported by another study which showed that despite smaller and longer Ca^2+^ transients, RyR content and activity was intact in failing canine and human hearts [179]. Nonetheless, it should be noted that studies using other models of HF have found ineffective coupling to contribute to the pathology [187–189].

As previously mentioned, SR Ca^2+^ uptake by SERCA is the primary pathway involved in lowering [Ca^2+^]_cyt_ and inducing relaxation of the myocardium during diastole. Several studies have shown that SERCA Ca^2+^ uptake is reduced in failing hearts from both human and animal models [177,179,181,185,190,191]. In theory, reduced SERCA activity could be due to either decreased SERCA content or increased PLN content leading to greater inhibition of SERCA. Interestingly, reductions in both SERCA and PLN content are consistently observed in HF [179,185,190,192,193], which translates to either unchanged or decreased SERCA:PLN ratios (i.e., greater inhibition of SERCA by PLN). Another reason behind the lower Ca^2+^ uptake in HF could be decreased phosphorylation of PLN leading to increased inhibition of SERCA. Although results have varied across models of HF, there is good evidence to suggest that PLN phosphorylation is probably reduced [194–196]. This may be surprising given that CAMKII expression and activity have been found to be increased in HF [197]; however, given that PLN phosphorylation status is dependent on a balance of kinase and phosphatase activity, this can be explained by increased protein phosphatase 1 (PP1) activity [198,199]. With regards to other studies which show contrasting results, the stage in the progression of HF at which phosphorylation of PLN was assessed can impact the results [194,195]. Regardless of changes in the levels of the aforementioned proteins observed across different models, Ca^2+^ uptake into the SR is reduced.

NCX, being the second major contributor in the myocardium to the transport of Ca^2+^ out of the cytosol, plays a compensatory role in HF. In the face of decreased SERCA content, NCX content, and accordingly its activity, is found to be increased in HF [192,200,201]. A study in failing canine cardiomyocytes found that while SERCA levels decreased by approximately 28%, NCX levels increased by 104% [192]. In order to assess the contribution of SERCA and NCX to Ca^2+^ removal during relaxation, the authors first inhibited SERCA and found the time it takes to reach 50% of maximal force during relaxation was significantly greater (i.e., slower relaxation) in the nonfailing hearts compared with the failing hearts indicating that SERCA has a greater contribution to relaxation in these cells. They then inhibited NCX by placing the cells in a Na^+^-free solution, which increased the time to 50% maximal force during relaxation in both sets of cardiomyocytes but only up to 30% in normal cells and up to 97% in failing cells, indicating that NCX contributes more to relaxation in the failing cells. More recently, a study using a guinea pig HF model found that while the contribution to Ca^2+^ removal by SERCA decreased by 28% that of NCX increased by 63% [202]. Although this compensation of increased NCX content helps to remove [Ca^2+^]cyt, it can actually increase the probability of arrhythmogenic events. In failing hearts, with increasing frequency of stimulation, the intracellular Na^+^ ([Na^+^]_cyt_) levels rise [186,203], due to increased activity of the Na^+^/H^+^ exchanger on the sarcolemma [204] and reduced Na^+^/K^+^ ATPase activity [202] causing increased reverse-mode NCX activity [186,205]. Under β-adrenergic stimulation, the rise in NCX-reverse current would increase [Ca^2+^]_cyt_ and SERCA uptake of Ca^2+^ into the SR would also increase leading to SR Ca^2+^ overload [186,205]. Therefore, the SR Ca^2+^ overload, alongside an increased membrane potential due to reduced inward rectifier K^+^ channel current, would increase the susceptibility to arrhythmias [186,205].

Abnormalities in Ca^2+^ handling in HF are not isolated to cardiac muscle but have similarly been found in skeletal muscle. Several animal studies have shown abnormalities in Ca^2+^ handling in skeletal muscle with HF, including reduced SERCA1 and 2 content [206], lower Ca^2+^ uptake [207], and lower Ca^2+^ release [208], which impairs muscle force generation capacity and increases fatiguability [208–210]. Middlekauff and colleagues (2012) sought to examine if such skeletal muscle Ca^2+^ handling abnormalities also occurred in humans with HF and whether oxidative stress may be involved [211]. Analyses of *vastus lateralis* biopsy samples revealed lower DHPR and SERCA2a content alongside lower phosphorylated PLN in advanced HF patients compared with healthy controls; however, there were no differences in markers of oxidative stress [211]. In cardiac muscle, chronic augmentation of sympathetic nerve activity leads to increased CAMKII expression and activity resulting in a phenomenon known as excitation transcription coupling whereby CAMKII acts as a long-term regulator of hypertrophic genes and Ca^2+^ handling proteins [212,213]. Thus, Middlekauf and colleagues postulated, because skeletal muscle is also subject to elevated sympathetic activity, that excitation-transcription coupling alongside increased PP1 activity may also be at play in this tissue causing the Ca^2+^ handling abnormalities observed. Therefore, the Ca^2+^ dysregulation seen in both tissues are mirroring each other likely due to the same mechanism.

### Altered redox regulation of SERCA in disease

As mentioned earlier, SERCAs are redox-sensitive proteins that are activated by low levels of ROS and RNS, but they are also highly susceptible to oxidative damage and inactivation by high levels of ROS and RNS (i.e., oxidative stress) [153,154,214–216]. Cohen and colleagues have shown that in disease, irreversible oxidation of key thiols, specifically cysteine-674, prevents activation of SERCAs through redox signaling mechanisms [87,217,218]. Several studies have also noted increased oxidation and nitration of SERCA in aged skeletal muscle [155,214,219], which we have shown results in the loss of redox control of SERCA activity and expression [220]. However, our work showing that several SERCA-binding proteins, including heat shock protein 70, PLN, and SLN, can protect SERCA structure and function during cellular stress [152,159,221], suggests that these SERCA-binding proteins may play a crucial role in protecting cellular Ca^2+^ homeostasis and preserving cardiac and skeletal muscle function under conditions of chronic oxidative stress and disease.

## Conclusion

Maintenance of Ca^2+^ homeostasis is essential for healthy muscle. Within skeletal myofibers and cardiomyocytes, transient increases in [Ca^2+^]_cyt_ during E-C coupling must be rapidly lowered to re-establish resting concentrations and induce muscle relaxation, which is primarily accomplished by SERCA-mediated Ca^2+^ transport into the SR. During short contractions of fast-twitch rodent and amphibian myofibers, the cytosolic Ca^2+^ buffer PV also contributes to muscle relaxation by rapidly lowering [Ca^2+^]_cyt_. In the heart, Ca^2+^ extrusion from cardiomyocytes through the NCX contributes significantly to ventricular relaxation, and the PMCA and MCU also contribute to a very minor extent. Impairments in Ca^2+^ transport and dysfunctional Ca^2+^ homeostasis has consistently been shown to be involved with the pathogenesis and/or the progression of multiple myopathies within skeletal muscle and in HF. A better understanding of the function and regulation of muscle Ca^2+^ transport proteins can allow for greater insights into skeletal and cardiac muscle physiology and disease. Furthermore, this body of work can lead to novel approaches in the treatment of diseases involving Ca^2+^ dysregulation.

## Abbreviations

α-CLD: α-catenin-like domain
[Ca^2+^]_cyt_: cytosolic calcium concentration
[Na^+^]_cyt_: cytosolic sodium concentration
Ca^2+^: calcium
CAMKII: calcium/calmodulin protein kinase II
CBD1: calcium binding domain 1
CBD2: calcium binding domain 2
CICR: calcium-induced calcium release
CNM: centronuclear myopathy
DHPR: dihydropyridine receptor
DMD: Duchenne’s Muscular Dystrophy
DWORF: Dwarf Open Reading Frame
E-C Coupling: excitation–contraction coupling
HF: heart failure
HFpEF: heart failure with preserved ejection fraction
HFrEF: heart failure with reduced ejection fraction
HSP72: heat shock protein 72
ICT: intracellular calcium transient
LV: left ventricle
MCU: mitochondrial calcium uniporter
MLN: myoregulin
Na^+^: sodium
NCX: sodium (Na^+^)/calcium (Ca^2+^) exchanger
PI(3)P: phosphatidyl inositol (3) phosphate
PLN: phospholamban
PV: parvalbumin
RNS: reactive nitrogen species
ROS: reactive oxygen species
RyR: ryanodine receptor
SERCA: sarco(endo)plasmic reticulum Ca^2+^-ATPase
SLN: sarcolipin
SR: sarcoplasmic reticulum
*V*_max_: maximal rate
